# Current insights on the roles of gut microbiota in inflammatory bowel disease-associated extra-intestinal manifestations: pathophysiology and therapeutic targets

**DOI:** 10.1080/19490976.2023.2265028

**Published:** 2023-10-11

**Authors:** Yizhe Tie, Yongle Huang, Rirong Chen, Li Li, Minhu Chen, Shenghong Zhang

**Affiliations:** a Department of Gastroenterology, The First Affiliated Hospital, Sun Yat-sen University, Guangzhou, China; b Department of Clinical Medicine, Zhongshan School of Medicine, Sun Yat-Sen University, Guangzhou, China

**Keywords:** Inflammatory bowel disease, extra-intestinal manifestation, gut microbiota, gut barrier, translocation, molecular mimicry, metabolite, immunocyte, cytokine

## Abstract

Inflammatory bowel disease (IBD) is a chronic, recurrent inflammatory disease of the gastrointestinal tract. In addition to digestive symptoms, patients with IBD may also develop extra-intestinal manifestations (EIMs), the etiology of which remains undefined. The gut microbiota has been reported to exert a critical role in the pathogenesis of IBD, with a similar pattern of gut dysbiosis observed between patients with IBD and those with EIMs. Therefore, it is hypothesized that the gut microbiota is also involved in the pathogenesis of EIMs. The potential mechanisms are presented in this review, including: 1) impaired gut barrier: dysbiosis induces pore formation in the intestinal epithelium, and activates pattern recognition receptors to promote local inflammation; 2) microbial translocation: intestinal pathogens, antigens, and toxins translocate via the impaired gut barrier into extra-intestinal sites; 3) molecular mimicry: certain microbial antigens share similar epitopes with self-antigens, inducing inflammatory responses targeting extra-intestinal tissues; 4) microbiota-related metabolites: dysbiosis results in the dysregulation of microbiota-related metabolites, which could modulate the differentiation of lymphocytes and cytokine production; 5) immunocytes and cytokines: immunocytes are over-activated and pro-inflammatory cytokines are excessively released. Additionally, we summarize microbiota-related therapies, including probiotics, prebiotics, postbiotics, antibiotics, and fecal microbiota transplantation, to promote better clinical management of IBD-associated EIMs.

## Introduction

Inflammatory bowel disease (IBD) is a chronic, recurrent, progressive, destructive inflammatory disorder of the gastrointestinal tract.^1^ The rising incidence, especially in newly industrialized countries such as China, has caused a considerable burden on the society.^2^ Crohn’s disease (CD) and ulcerative colitis (UC) are the two major subtypes of IBD. The main clinical features of CD include diarrhea, abdominal pain, and weight loss, whereas UC is characterized by mucopurulent bloody stools.^3^ Currently, the most commonly accepted hypothesis regarding IBD etiology is that environmental factors acting on genetically susceptible populations result in a leaky gut barrier, microbiota dysbiosis, and ultimately overactivation of immune responses.^4^ In particular, the intestinal microbiota has been shown to take a critical position.^5^ Diet, 5-amino salicylic acid (5-ASA), corticosteroids, immunosuppressors, biologics, and small molecules are widely used for the management of IBD.^1,6^

Not only involving the gastrointestinal tract, IBD manifests in extra-intestinal organs in up to 50% IBD patients.^7^ These EIMs are defined as inflammations beyond the digestive tract in patients with IBD, which develop either as expansion of intestinal inflammatory responses or with similar genetic or environmental predisposition to IBD.^7^ Most EIMs develop in parallel with the disease activity of IBD and are associated with unfavorable prognosis, including medication escalation, surgery, and poor quality of life.^8^ Thus, EIMs have been placed in an increasingly important position for better management of IBD. However, the pathophysiology of EIMs remains unclear and generally include genetic susceptibility, environmental factors, dysregulated immune responses, and microbiota dysbiosis.^7^

Patients with IBD partly share genetic mutations, including that of nucleotide-binding oligomerization domain-containing protein 2 (NOD2)/caspase recruitment domain 15 (*CARD15*), with those who develop EIMs.^9^ In addition, common environmental factors, including higher exposure to smoking, have been reported in IBD and EIMs.^10^ Furthermore, a series of immunocytes, pro-inflammatory cytokines, and signaling pathways, such as interleukin (IL)-23 and IL-17, has been associated with the development of EIMs.^11^ Accordingly, the gut microbiota, which interacts with genetic susceptibility and environmental factors and is highly responsible for immune activation, is thus hypothesized to take an essential role in the pathogenesis of EIMs.

In this review, we first introduce the overall pathophysiology of EIMs. Next, we summarize gut dysbiosis in some common EIMs and further explore the essential roles of microbiota in the pathogenesis. Finally, we discuss the potential treatment options based on microbiota for IBD-associated EIMs.

## EIMs in IBD

According to the European Crohn’s and Colitis Organization, up to 50% of IBD patients experience at least one EIM.^7^ Among the diverse types of EIMs, musculoskeletal system (e.g., peripheral arthritis, axial spondyloarthropathy), mucocutaneous system (e.g., psoriasis), ocular system (e.g., uveitis), hepatobiliary tract (e.g., primary sclerosing cholangitis [PSC]) and oral cavity (e.g., periodontitis) are most frequently affected (Table 1). Other systems may also be involved, such as the cardiovascular system (e.g., ischemic heart disease), respiratory system (e.g., interstitial pneumonia), pancreas, and urogenital system (e.g., renal insufficiency).^8,33^ Typically, EIMs manifest after IBD, while nearly 25% patients develop EIMs before the occurrence of IBD, particularly uveitis and axial spondyloarthropathy.^34^

**Table 1.** Extra-intestinal manifestations of inflammatory bowel disease in adults: prevalence, susceptible groups and available treatments.

Extraintestinal Manifestations	Prevalence with IBD	Risk Association with CD/UC and Other EIMS	Association with IBD Activity	Available Treatment	Reference
**Musculoskeletal manifestations**					
Axial pondyloarthropathy	5% (range 1–46%).		Independent.	Physiotherapy; NSAIDs; MTX, 5-ASA; Biologics (IFX, ADA); Small molecules (tofacitinib).	^12–15^
Peripheral arthropathy	16% (range 1–43%).	More often in patients with CD; Higher risk in patients with IBD involved with colonic or perianal disease, uveitis, EN, PG and stomatitis.		5-ASA; NSAIDs; Steroid; MTX; Biologics (IFX, ADA, VDZ, UST).	^12,14,15^
Type 1	Type 1 ＞ Type 2.	Higher risk of EN and uveitis.	Parallel.	Management of IBD.	^13,16^
Type 2		Higher risk of uveitis.	Independent.		^13,16^
**Cutaneous manifestations**					
Psoriasis	3.8% (95%CI 3.1–4.6%).	More often in patients with CD.		VitD, Acitretin; Phototherapy, photochemotherapy; Steroid; Immunomodulator (MTX, CsA); Biologics (IFX, ADA, IL-23i).	^17,18^
Erythema nodosum	Up to 15%.	More often in patients with CD; Independently associated with PG, joint and ocular EIMs.	Parallel.	Management of IBD; Steroid; Immunomodulator; Biologics (anti-TNF).	^13,19–21^
Pyoderma gangrenosum	Up to 2.6%.	Higher risk in patients with ocular EIMs.		Steroid; Immunomodulator (e.g., CsA); Biologics (IFX, ADA, UST); Calcineurin inhibitors (tacrolimus).	^22,23^
**Ocular manifestations**					
Episcleritis	CD: .26%; UC: .21%.		Parallel.	Management of IBD; NSAIDs; Steroid.	^24–26^
Uveitis	.19–1.09%.	Higher risk in patients with CD.		Cycloplegics; Steroid; Immunomodulators; Biologics (anti-TNF).	^24,27^
**Hepatobiliary manifestations**					
PSC	2.16%.	More often in patients with UC.		No way to delay to liver transplantation, cancer, or death; Corticosteroids or immunomodulators, when sharing features with AIH.	^8,28^
**Oral manifestations**					
Periodontitis	37.5%.	Higher prevalence and severity in patients with UC; Ocular EIMs.	Parallel.	Scaling and root planning; Chlorhexidine; ASA; Probiotics, antibiotics, postbiotics; Laser therapy, surgery.	^29–32^

Abbreviations: IBD, inflammatory bowel disease; CD, crohn’s disease; UC, ulcerative colitis; EIMs, extra-intestinal manifestations; EN, erythema nodosum; PG, pyoderma gangrenosum; NF-κB, nuclear factor-κB; VitD, vitamin D; ASA, amino salicylic acid; NSAIDs, non-steroidal anti-inflammatory drugs; anti-TNF, anti-tumor necrosis factor; IFX, infliximab; ADA, adalimumab; UST, ustekinumab; VDZ, vedolizumab; IL-17i, interleukin-17 inhibitor; IL-23i, interleukin-23 inhibitor; MTX, methotrexate; CsA, cyclosporin A; AIH, autoimmune hepatitis.

The occurrence of EIMs poses a great impact on the quality of life of patients with IBD. Most EIMs develop in parallel with disease activity (Table 1) and may indicate a higher risk of treatment escalation.^35^ Furthermore, the presence of EIMs could affect the original therapeutic approach. For example, vedolizumab, a highly gut-selective biological agent, should be cautiously administered in cases of EIMs. Confronted with EIMs independent of IBD disease activity, the benefit of other biologics such as infliximab would overwhelm that of vedolizumab.^36^

The remarkable relationship between EIMs and IBD leads to the hypothesis that their underlying pathophysiological mechanisms share certain similarities. An overlap of susceptible gene loci between IBD and EIMs has been reported, and a higher concordance rate for EIMs has been observed in parent-child pairs and sibling pairs.^33,37^ Mutations in *CARD15* contribute to the intracellular persistence of pathogens and a higher risk of joint inflammation. More specifically, encoded by *CARD15*, NOD2 recognizes bacterial cell wall components and activates nuclear factor-κB (NF-κB), which regulates the release of pro-inflammatory cytokines.^9^ Additionally, a more frequent exposure to environmental factors such as smoking has been correlated with a higher prevalence of EIMs, while cessation is associated with a lower prevalence.^10^ Gut dysbiosis has also been reported in EIMs (Table 2), in which microbiota, antigens, and toxins might translocate via the impaired intestinal barrier into other extra-intestinal sites, leading to the overactivation of inflammatory responses. As a result, inflammatory signaling pathways are up-regulated with aberrant immunocyte homing and excessive pro-inflammatory cytokine secretion. For instance, the tumor necrosis factor (TNF)-NF-κB pathway is up-regulated in patients in the context of erythema nodosum and pyoderma gangrenosum; correspondingly, anti-TNF therapy has been proven effective in the management of such patients.^70^ Moreover, the expression of mucosal vascular addressin cell adhesion molecule 1 in the liver and bone marrow induces aberrant lymphocyte homing, triggering inflammation at these extra-intestinal sites.^11,71^

**Table 2.** Changes and roles of common gut microbiota that contributes to IBD-associated EIMs.

Gut Microbiota	Changes in IBD	Relevant Mechanisms in EIMs	Reference
**(A) SpA**			
Bifidobacterium	Increase (IBD).	Participate in the production of SCFA (e.g., propionate).	^38,39^
Ruminococcus gnavus	Increase (IBD).	Degrade mucin and destroy the gut barrier; Produce pro-inflammatory polysaccharide, inducing TNF-α secretion of dendritic cells.	^40–42^
Klebsiella	Increase (UC).	Reduce tight junction-associated proteins; Activate NF-κB and promote the secretion of pro-inflammatory IL-1, IL-6 and TNF-α.	^43,44^
Escherichia coli	Increase (IBD).	Promote the expression of zonulin, down-regulating tight junction proteins.	^38,45–47^
Roseburia	Decrease (IBD).	Participate in the production of SCFA (e.g., butyrate).	^48,49^
Faecalibacterium prausnitzii	Decrease (IBD).	Participate in the production of SCFA (e.g., butyrate); Secrete metabolites, inhibiting NF-κB activation and IL-8 production.	^48,49^
**(B) Psoriasis**			
Ruminococcus	Decrease (IBD).	Correlate with secondary bile acid; Participate in the production of SCFA (e.g., propionate).	^39,48,52–54^
Akkermansia muciniphila	Decrease (IBD).	Improve the gut barrier; Participate in the production of SCFA (e.g., propionate); Induce IgG1 production and antigen-specific T cell responses.	^39,40,52,55,56^
Faecalibacterium prausnitzii	Decrease (IBD).	Participate in the production of SCFA (e.g., propionate); Secrete metabolites, inhibiting NF-κB activation and IL-8 production.	^38,39^
Saccharomyces cerevisiae	Decrease (IBD).	Restore barrier function via inhibiting the expression of pore-forming claudin-2.	^58–60^
**(C) Uveitis**			
Clostridium	Decrease (IBD).	Participate in the production of SCFA (e.g., propionate); Participate in the de-conjugation and the de-hydroxylation in bile acid metabolism.	^38,39^
Ruminococcus	Decrease (IBD).	Correlate with secondary bile acid; Participate in the production of SCFA (e.g., propionate).	^39,48,53,54,62^
Akkermansia muciniphila	Decrease (IBD).	Improve the gut barrier; Participate in the production of SCFA (e.g., propionate); Induce IgG1 production and antigen-specific T cell responses.	^39,40,55,56,62^
Bacteroides	Decrease (IBD).	Degrade mucin, improve the epithelial tight junction barrier function; Participate in the production of SCFA (e.g., propionate); Inhibit NF-κB and reduce pro-inflammatory IL-8, TNF-α.	^38,39,62,63^
Lachnospira	Decrease (IBD).	Participate in the production of SCFA (e.g., butyrate).	^38,62^
Faecalibacterium prausnitzii	Decrease (IBD).	Participate in the production of SCFA (e.g., propionate); Secrete metabolites, inhibiting NF-κB activation and IL-8 production.	^38,39^
**(D) PSC**			
Veillonella parvula	Increase (CD).	Participate in the production of SCFA (e.g., propionate).	^39,48,64^
Enterococcus faecalis	Increase (IBD).	Produce matrix metalloproteinase, impairing mucosal integrity.	^38,65,66^
Klebsiella	Increase (UC).	Induce pore formation in the gut epithelium.	^43,67^
Ruminococcus	Decrease (IBD).	Correlate with secondary bile acid; Participate in the production of SCFA (e.g., propionate).	^39,48,54,64^
Coprococcus	Decrease (IBD).	Participate in the production of SCFA (e.g., propionate).	^39,48,64^
Faecalibacterium prausnitzii	Decrease (IBD).	Participate in the production of SCFA (e.g., propionate); Secrete metabolites, inhibiting NF-κB activation and IL-8 production.	^39,48,51,64^
Saccharomyces cerevisiae	Decrease (IBD).	Restore barrier function via inhibiting the expression of pore-forming claudin-2.	^58,60,68^
**(E) Periodontitis**			
Escherichia	Increase (IBD).	Promote the expression of zonulin, down-regulating tight junction proteins.	^38,45,47,69^
Coprococcus	Decrease (IBD).	Participate in the production of SCFA (e.g., propionate).	^39,48,64,69^
Lachnospira	Decrease (IBD).	Participate in the production of SCFA (e.g., butyrate).	^38,62,69^

Abbreviations: IBD, inflammatory bowel disease; CD, crohn’s disease; UC, ulcerative colitis; EIMs, extra-intestinal manifestations; SpA, spondyloarthropathy; PSC, primary sclerosing cholangitis; SCFA, short-chain fatty acid; TNF-α, tumor necrosis factor-α; NF-κB, nuclear factor-κB; IL-8, interleukin 8; IgG, immunoglobulin G.

## Mechanisms linking gut dysbiosis to IBD-associated EIMs

As is widely accepted, genes, environment, microbiota, and immune responses contribute to the development of IBD.^72,73^ Among these, gut dysbiosis plays an essential role. This may be corroborated by a finding that genetically susceptible mice raised in a germ-free environment manifest less colitis, whereas re-exposure to pro-inflammatory microbiota induces gut inflammation.^74^ In patients with IBD, gut dysbiosis such as increased *Bifidobacterium, Enterococcus faecalis, Escherichia coli, Klebsiella, Ruminococcus gnavus, Veillonella parvula* and decreased *Akkermansia muciniphila, Bacteroides, Clostridium, Coprococcus, Faecalibacterium prausnitzii, Lachnospira, Roseburia, Saccharomyces cerevisiae*, have been reported.^38,40,43,48,58^ The dys-regulated microbiota disrupts the gut barrier, disturbs the metabolism of bile acids (BA) and short-chain fatty acids (SCFA), promotes the hyper-activation of immunocytes and the over-production of pro-inflammatory cytokines, thus contributing to the development of IBD.^61^

**Figure 1.**
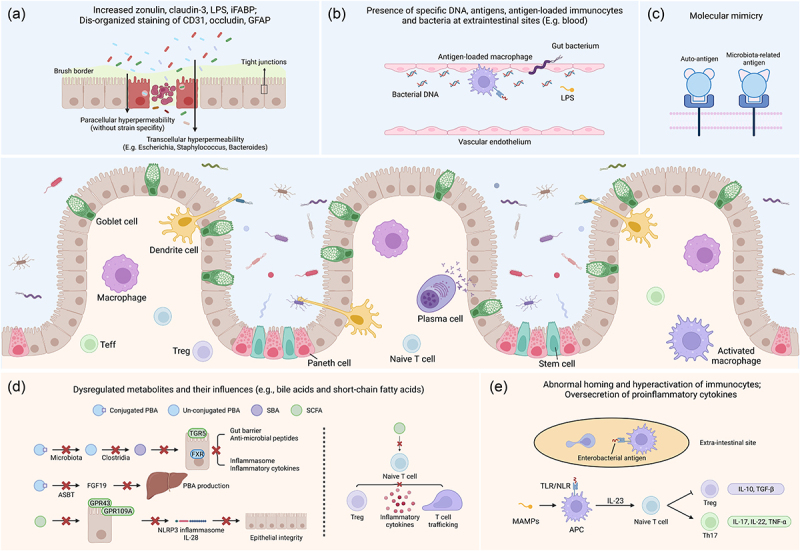
Roles of gut microbiota in the pathophysiology of IBD-associated EIMs. Generally, the intestinal epithelium is composed of enterocytes (the majority, defense and nutrition), goblet cells (scattered, secrete mucus), Paneth cells (crypt base, secrete anti-microbial peptides and regulate stem cells), stem cells (crypt base, proliferate), etc. Macrophages and DCs recognize microbial antigens, secrete pro-inflammatory cytokines or present antigens to other lymphocytes (e.g., naïve T cells, plasma cells), responsible for immune over-activation in the development of IBD and EIMs. (a) impaired gut barrier: transcellular hyper-permeability and paracellular hyper-permeability appear as a result of gut dysbiosis, respectively contributing to specific bacteria internalization and nonspecific microbiota translocation. Elevated expression of zonulin induces a down-regulation of occludin and E-cadherin. (b) translocation: increased levels of gut microbiota, relevant antigens (e.g., DNA) or toxins (e.g., LPS), antigen-loaded immunocytes are detected in the serum of patients with EIMs. (c) molecular mimicry: some bacterial antigens (e.g., Klebsiella nitrogenase) share similar epitopes with self-antigens (e.g., HLA-B27) and induce activation of auto-reactive immunocytes. (d) microbiota-related metabolites: dysregulated metabolism of BAs, SCFAs, MCFAs, etc., results in the impaired mucosa integrity, attenuation of anti-inflammatory response and promotes T cell trafficking toward extra-intestinal sites. (e) immunocytes and cytokines: aberrant expression of cell adhesion molecules and interaction with antigen-loaded macrophages are responsible for abnormal homing of lymphocytes. Activated APCs also favor differentiation of naïve T cells in the direction of pro-inflammatory Th17 cells. Treg, regulatory T cell; teff, effector T cell; LPS, lipopolysaccharide; iFABP, intestinal fatty acid binding protein; GFAP, glial fibrillary acidic protein; PBA, primary bile acid; SBA, secondary bile acid; SCFA, short-chain fatty acid; TGR5, transmembrane G protein-coupled receptor 5; FXR, farnesoid X receptor; ASBT, apical sodium-dependent bile acid transporter; FGF19, fibroblast growth factor 19; GPR43, G protein-coupled receptor 43; GPR109A, G protein-coupled receptor 109A; NLRP3, NOD-, LRR- and pyrin domain-containing 3; MAMPs, microbe associated molecular patterns; TLR, toll-like receptor; NLR, NOD-like receptor; APC, antigen-presenting cell; TNF-α, tumor necrosis factor-α; TGF-β, tumor growth factor-β.

Similarly, in the context of EIMs, animal models that tend to spontaneously develop spondylarthritis (SpA)^75^ or uveitis^76^ exhibit less severity when raised in germ-free conditions. However, once the animals are re-exposed to a specific pathogen-free environment or microbial components, the EIMs manifest, which indicates microbiota’s essential effects in EIMs.^75,76^ Moreover, considering that multiple studies have observed the similar pattern of gut dysbiosis in IBD and various types of EIMs (Table 2), it is hypothesized that gut microbiota might exert a significant role with regard to the pathophysiology of EIMs. Afterwards, some of the underlying mechanisms between gut microbiota and EIMs has been discovered, which could be briefly summarized into five paths: 1) impaired gut barrier: dysbiosis induces pore formation in the intestinal epithelium, and activates pattern recognition receptors to promote local inflammation; 2) microbial translocation: intestinal pathogens, antigens, and toxins translocate via the impaired gut barrier into extra-intestinal sites; 3) molecular mimicry: certain microbial antigens share similar epitopes with self-antigens, inducing inflammatory responses targeting extra-intestinal tissues; 4) microbiota-related metabolites: dysbiosis results in the dysregulation of microbiota-related metabolites, which could modulate the differentiation of lymphocytes and cytokine production; 5) immunocytes and cytokines: immunocytes are over-activated and pro-inflammatory cytokines are excessively released (Figure 1).^7^

### Impaired gut barrier

The gut barrier is primarily composed of digestive juice (e.g., gastric acid), commensal microbiota, antimicrobial peptides, epithelial cells, and local immunocytes.^77^ Brush border and tight junctions of epithelial cells respectively serve as the transcellular barrier and the paracellular barrier against intestinal flora.^78^ In terms of IBD and EIMs, an elevated abundance of mucin-degrading bacteria (e.g., *R. gnavus*) contributes to the disruption of epithelial integrity.^41,79^ Zonulin and enzymes released by gut microbiota down-regulate the expression of tight junction proteins, resulting in the impaired intestinal barrier.^45,65^ Furthermore, transcellular hyper-permeability occurs through the internalization of specific bacterial strains such as *Escherichia coli* and *Bacteroides vulgatus*.^78^ Subsequently, paracellular hyper-permeability occurs, leading to nonspecific penetration of the microbiota.^78^ In this way, a vicious cycle is created.

In fact, down-regulated tight junction proteins and elevated zonulin have been reported in patients with ankylosing spondylitis (AS).^45^ Additionally, microbiota isolated from ileal biopsies, such as *E. coli*, which is enriched in patients with IBD and those with SpA,^38,46^ promote the expression of zonulin in cultured epithelial cells.^45,47^ Elevated zonulin further down-regulates the levels of occludin, E-cadherin, and other tight junction proteins, thus impairing the gut vascular barrier.^45^ Correspondingly, after applying antibiotics, the gut microbiome and the expression of tight junction proteins are restored.^45^

Elevated intestinal fatty acid-binding protein, a predictor of the impaired gut barrier, seems positively correlated with disease severity in patients with psoriasis.^80^ Enriched *Campylobacter* spp. and decreased *A. muciniphila* appear in patients with IBD psoriasis, and correlated with the hyper-permeability of gut mucosa.^40,52,55,81,82^

In patients with uveitis, the intestinal epithelium is disorganized. In animal models of autoimmune uveitis, the course of impaired intestinal permeability coincides with the extent of gut dysbiosis and the degree of intestinal permeability corresponds with that of ocular inflammation.^83,84^

In the context of IBD and PSC, expansion of *E. faecalis* promotes matrix metalloproteinase (e.g., gelatinase) production, degrading E-cadherin, down-regulating trans-epithelial electrical resistance, and impairing mucosal integrity.^38,65,66^ Furthermore, enriched *Klebsiella pneumoniae* could trigger pore formation in the gut epithelium in vitro.^43,67^

Notably, in animal models of periodontitis, berberine contributes to an increased abundance of butyrate-producing gut microbiota (e.g., *Roseburia*), which is reduced in IBD patients.^38,85^ Restoration of the gut microbiota improves the gut barrier, lowers the circulatory endotoxin levels, and ameliorates periodontitis, indicating the potential of gut dysbiosis in the pathogenesis of periodontal disease.^85^

### Translocation of bacteria, antigens, and toxins

Penetrating via the impaired gut barrier, harmful gastrointestinal bacteria or relevant toxins and antigens are translocated via the blood or the lymphatics into extra-intestinal sites, including the synovial tissue.^86,87^ Sometimes, such bacteria, antigens, or toxins can be engulfed and later presented on the surface by immunocytes such as dendritic cells (DCs) and macrophages.^86,88^ These engulfed bacteria might remain quiescent, or their lysed components (e.g., lipopolysaccharides [LPS]) are released to induce chronic inflammation. Moreover, lymphatic expansion has been confirmed in the context of gastrointestinal inflammation,^86^ indicating its potential role in the translocation of the gut microbiota.

Although no living microbiota has been isolated from the joint fluid, antigens related to *Yersinia enterocolitica* and *Salmonella enteritidis* have been observed in patients with reactive arthritis. Similarly, their DNA have been detected in the intestinal samples and mesenteric lymph nodes (MLN) of patients with CD.^89,90^ In addition, serum bacterial products, such as LPS and intestinal fatty acid-binding proteins, are increased in patients with IBD-associated SpA.^91^ Artificial injection of peptidoglycan-polysaccharide complexes successfully induces erosive peripheral arthritis in animal models,^92^ further indicating a significant role of the gut microbiota.

Elevated serum levels of gut bacterial DNA (e.g., *E. coli*) have also been reported in patients with psoriasis.^87^ Methylated DNA segments (e.g., CpG) might act as microbe-associated molecular patterns (MAMPs) to activate immunocytes, such as DCs, thus promoting inflammation in the skin. Accordingly, a higher quantity of microbial DNA typically corresponds with increased circulatory pro-inflammatory cytokine levels (e.g., IL-1β, IL-6, IL-12, TNF-α, and IFN-γ) via interacting with Toll-like receptor 9.^87^

Although no living microbiota or relevant antigens have been detected in uvea of uveitis patients,^83^ artificial injection of the group A *streptococcus* peptidoglycan-polysaccharide complex, which is elevated in IBD patients, successfully induced bilateral uveitis and polyarthritis in Lewis rats.^38,92^

Enriched *Enterococcus gallinarum*, and *K. pneumoniae*, have been observed in patients with IBD and PSC.^43,67,93^ These bacteria have also been isolated from the MLN of gnotobiotic PSC/UC mice, which is derived from fecal samples from PSC/UC patients.^67^

Similarly, oral – gut microbiota axis has been proposed to participate in both oral and gastrointestinal inflammation. As has been reported, *Klebsiella* and *Enterobacter* spp., originally isolated from the oral cavity, effectively elicit the activation of T helper cells and inflammasome, contributing to the development of colitis.^94,95^ In addition, elevated circulatory LPS has been reported in animal models of periodontitis. Interestingly, administration of berberine significantly reduces LPS levels, preventing hematogenous translocation of gut pathogens, and thus improving the degree of alveolar bone loss.^85^

### Molecular mimicry

Certain bacterial antigens might share similar amino acid sequences or molecular structures with self-antigens, such as major histocompatibility complex molecules, inducing over-activation of auto-reactive immunocytes targeting human tissues.^96^

In the context of SpA, antigens related to *Bacillus megaterium*, *E. coli*, *Klebsiella* (nitrogenase), *Pseudomonas aeruginosa*, and *Salmonella typhimurium* share similar amino acid sequences (e.g., LRRYLENGK) with human leukocyte antigen class I molecule B27 (HLA-B27).^43,96,97^ Consequently, T cells are highly activated in response to macrophages presenting peptides of either bacterial antigens or the endogenous HLA-B27 turnover product.^97^ *Chlamydia trachomatis*, another arthritis related microbiota, expresses a specific DNA primase that shares a similar sequence with a dodecapeptide of HLA-B27.^98^ Patients with IBD suffered a high risk of *Chlamydia trachomatis* infection,^99^ which might contribute to the development of SpA. Similarities also exist between *K. pneumonia* nitrogenase reductase and HLA-B27 (QTDRED),^100^ *K. pneumonia* pullulanase and HLA-B27 (DRED), *K. pneumonia* pullulanase and collagens type I, III and IV (“Gly-X-Pro”),^101^ which reportedly participate in the development and progression of IBD and SpA.

### Microbiota-related metabolites

Gut microbiota participates in the production and excretion of various metabolites. Therefore, as a result of gut dysbiosis, microbiota-related metabolites are dys-regulated, leading to disruption of their functions in maintaining the gut barrier, inhibiting microbiota translocation, modulating activation of inflammasomes or other inflammation-related signaling pathways, and directing differentiation and trafficking of immunocytes.^102^

#### Bile acids

Primary bile acids (PBAs), primarily including chenodeoxycholic acid (CDCA) and cholic acid (CA), are produced in the liver. PBAs are conjugated to taurine or glycine, which are subsequently released to the intestinal lumen. Most are re-absorbed in the distal ileum via the apical sodium-dependent bile acid transporter, while the remainder is further metabolized in the colon. This process involves deconjugation via bile salt hydrolase, and subsequent de-hydroxylation by various microbiota, e.g., *Bacteroides*, *Clostridium*, and *F. prausnitzii*, ultimately generating secondary bile acids (SBAs, primarily including lithocholic acid [LCA], deoxycholic acid [DCA]).^103^ Bile acids act by interacting with transmembrane G protein-coupled receptor 5 (TGR5), farnesoid X receptor (FXR), etc. Notably, CDCA has the highest affinity for FXR, while SBAs have a high affinity for TGR5.^104,105^ Activation of FXR prevents gut hyper-permeability, inhibits bacterial overgrowth and translocation, promotes antimicrobial peptide production, and down-regulates NF-κB associated TNF-α, IL-1β, and IL-6 production.^106,107^ Meanwhile, TGR5 activation modulates an M2-dominant anti-inflammatory macrophage phenotype transformation, inhibits NF-κB associated pro-inflammatory cytokine production via the cyclic adenosine monophosphate (cAMP)-protein kinase A (PKA) pathway, and blocks the production of IL-1β and the Nod-like receptor protein 3 (NLRP3) inflammasome. Hence, FXR and TGR5 activation both alleviate intestinal inflammation.^108,109^ Furthermore, SBAs promote the differentiation of anti-inflammatory regulatory T cells (Tregs).^108,110,111^ In IBD, decreased *Clostridium*, and *F. prausnitzii* disturb the de-conjugation and de-hydroxylation processes, resulting in increased PBA and decreased SBA levels in serums and feces.^38,103,112,113^

In uveitis, SBAs are down-regulated and inhibit DC-secreted pro-inflammatory cytokines such as IL-1β, IL-6, IL-12, and TNF-α via the TGR5-cAMP-PKA-NF-κB signaling pathway, further directing the differentiation of naïve T cells in experimental autoimmune uveitis.^114^

For patients with IBD-associated PSC, total BAs are significantly down-regulated in fecal samples, with non-significantly increased conjugated BAs. Moreover, levels of SBAs are negatively correlated with disease activity.^115^ Additionally, the abundances of *Clostridium* spp. and *Ruminococcus* spp. are decreased in patients with PSC, which contribute to deconjugation and dihydroxylation of BAs and thus bio-transformation of PBAs to SBAs.^64,103,116^

For patients with periodontitis, significantly increased conjugated BAs are detected in gingival tissues, which are positively correlated with periodontitis status.^117^ Other forms of BAs, i.e., primary and secondary, conjugated and unconjugated, are also up-regulated in patients with periodontitis.^117^

#### Short-chain fatty acids

Short-chain fatty acids (SCFAs), primarily including acetate, propionate, and butyrate, are synthesized from dietary fibers during the gut microbe metabolism. After interacting with G protein-coupled receptor 43 (GPR43) or GPR109A, SCFAs regulate the activation of the NLRP3 inflammasome and production of IL-18, participating in the repair of the intestinal barrier.^118^ Butyrate also modulates the expression of MUC genes in goblet cells and thus the properties of mucus, enhancing the barrier function.^119^ Most importantly, SCFAs inhibit the activity of histone deacetylase, promote the differentiation of naïve T cells toward Tregs instead of T helper type 17 (Th17) cells, and regulate the circulation of T cells toward extra-intestinal sites such as the eyes.^102,120^ Modulation of pro-inflammatory cytokine secretion and B cell responses, such as plasma cell differentiation and IgG production, has also been observed.^121^ Moreover, SCFAs reportedly suppress DC maturation, down-regulate DC-secreted chemokines such as C-C chemokine ligand 5, and up-regulate anti-inflammatory IL-10 in DCs to further promote Treg function.^121^ The anti-inflammatory effects of macrophages (e.g., IL-10 production) are also strengthened following SCFA supplementation.^121^

For patients with IBD, SpA, psoriasis, uveitis, PSC, or periodontitis, the abundance of butyrate-producing bacteria (e.g., *A. muciniphila, Clostridium XIVa, Coprococcus, F. prausnitzii, Lachnospira, Roseburia spp. and Ruminococcus spp*.) was reduced.^38,50,52,57,62,64,69,122^ Meanwhile, fecal samples from patients with arthritis exhibit reduced butyrate levels.^123^ Butyrate was also found to inhibit the proliferation of antigen-specific B lymphocytes and cytokine production of natural killer T cells in rodents. Hence, butyrate down-regulates the degree of inflammation and tissue degradation of the joint.^124^

#### Other metabolites

Other microbiota-related metabolites have been linked to the pathogenesis of EIMs. Tryptophan, normally metabolized by microbiota in the indole pathway, activates the aryl hydrocarbon receptor, promotes Treg differentiation, and participates in the IL-10 and IL-22 pathways.^102^ Decreased serum tryptophan levels and increased serum kynurenine levels have been observed in patients with AS.^125^ Tryptophan metabolism has also been markedly disrupted (e.g., up-regulated L-kynureninase) in psoriasis.^126^

Similarly, patients with IBD-associated rheumatoid arthritis exhibits further microbial tyrosine degradation.^127^ In addition, lower levels of IgA and medium-chain fatty acids have been reported in the stool of patients with psoriasis, which is correlated with the decreased abundance of *Akkermansia*, *Coprococcus*, and *Ruminococcus*.^128^ Enriched phenol and p-cresol suppress the expression of keratin 10 and dys-regulate the differentiation of keratinocytes as well as the skin barrier function.^129^ Moreover, trimethylamine N-oxide is elevated in the blood of patients with psoriasis, inducing M1-type polarization of macrophages, promoting inflammation, and increasing the risk of psoriasis comorbidities.^130,131^ Decreased circulatory branched-chain amino acids and vitamin B6 have also been demonstrated in patients with PSC.^132^

### Immunocytes and cytokines

In addition to hyper-activation of immunocytes, such as macrophages, DCs, and lymphocytes, gut dysbiosis also participates in the aberrant homing of immunocytes toward extra-intestinal sites and the over-secretion of pro-inflammatory cytokines such as IL-1β, IL-6, and IL-17. MAMPs such as LPS stimulate Toll-like receptors and nucleotide-binding oligomerization domain (NOD)-like receptors.^133^ Activation of Toll-like receptors and NOD-like receptors result in the differentiation of Th1 and Th17 cells, and the production of pro-inflammatory cytokines (TNF, IL-17, IL-22, etc.), which in turn worsens intestinal hyper-permeability and translocation.^134^

In the context of SpA and psoriasis, once stimulated by dysbiosis, DCs and macrophages secrete IL-23, activating type 3 innate lymphoid cells and Th17 cells to produce and release pro-inflammatory cytokines, including IL-17, IL-22, and TNF-α.^11,135,136^ Systemic administration of β-1,3-glucan (a fungal element) reportedly promotes mucosal dysfunction and cytokine secretion, leading to the SpA syndrome in an IL-23/IL-17 dependent pattern (i.e., IL-23 amplifies endoplasmic reticulum stress).^137^ Similarly, the gut microbiota from arthritis-susceptible mice significantly up-regulate Th17 cells and down-regulate Tregs and DCs in the spleens of germ-free mice.^138^ The introduction of segmented filamentous bacteria (SFB) could induce arthritis driven by Th17 cells, while neutralization of IL-17 successfully blocks the formation of germinal centers and the progression of arthritis in specific-pathogen-free K/BxN mice.^139^ Moreover, SFB induces T follicular helper cell differentiation in Peyer’s patches via DC-mediated inhibition of the IL-2-related pathway and directs T follicular helper cell trafficking toward systemic lymphoid tissues responsible for auto-antibody production, which might contribute to SFB-induced arthritis.^140^ Besides, enrichment of IgA-coated adherent-invasive *E. coli* (AIEC) was observed in feces of CD patients with SpA compared to those with CD alone, in which subsequent functional analysis showed that AIEC trigger Th17 cells activation to increase systemic immune responses, including joint and intestinal inflammation.^141^ *Parabacteroides distasonis*, another intestinal bacterium reduced in patients with rheumatoid arthritis, could inhibit the expression of auto-antibodies and pro-inflammatory cytokines (IL-1β, IL-6, IL-17A, and TNF-α), increase the mRNA levels of anti-inflammatory cytokines (IL-10), reverse the Th17/Treg imbalance in MLN of mice with arthritis, and even induce M2-type polarization of macrophages via its products such as iso-lithocholic acid and 3-oxo-lithocholic acid.^142^ Similarly, enriched *Prevotella* spp. have been detected in patients with rheumatoid arthritis, while administration of *P. copri* exacerbates joint inflammation in animal models of arthritis via over-producing succinate, and up-regulating pro-inflammatory responses in macrophages.^143^ A special group of macrophages that are enriched in the gut mucosa and synovium, expresses the scavenger receptor CD163 and present bacterial antigens, potentially activating the corresponding T cell groups, and contributing to joint inflammation.^88^ In addition, another T cell group specific to gut microbiota-related antigens has been isolated from the joint fluid and synovium of patients with reactive arthritis.^144^ CD4^+^ T cells might become activated in the gut under the stimulus of macrophage-presenting bacterial antigens, travel into the joints, and induce inflammatory responses. The trafficking of such immunocytes depends on the selectively increased expression of vascular adhesion protein-1, CD44, very late antigen-4, and integrin α4β7, as well as the up-regulation of mucosal vascular addressin cell adhesion molecule 1 in the bone marrow.^11,96^ Correspondingly, a high expression of IL-17 has been detected in the gut and synovial fluid of patients with SpA. Downstream TNF-α induces synovial fibroblasts to release more matrix-degrading metalloproteinases and destroy tissues in the joints.^96^

In animal models of uveitis, the intestinal microbiota is sufficient to activate T cells specific for disease development, regardless of the endogenous retinal auto-antigens (i.e., interphotoreceptor retinoid-binding protein). However, after heat inactivation or pre-treatment with Proteinase K, the intestinal contents lose the ability to induce uveal inflammation.^76^ Furthermore, with the aid of specific transgenic mouse models, lymphocytes originally in the colon have been detected in the eye, supporting that lymphocyte trafficking may participate in uveitis progression.^120^

In patients with IBD-associated PSC, increased abundance of *Sphingomonas* sp. and *Veillonella* sp. is reported. Consequently, more amino oxidases are produced, among which vascular adhesion protein-1 promotes the trafficking and the adherence of original gut lymphocytes toward the hepatic endothelium.^122,145^ IL-17-producing lymphocytes, such as Th17 cells, are also increased in the peri-ductal area. Moreover, increased recruitment of macrophages in the liver is associated with gut dysbiosis in dextran sodium sulfate-administered animal models of PSC.^146^

As for periodontitis, it has been reported that translocation of *Porphyromonas gingivalis* from oral cavity to the intestinal tract could enhances Th17 cells differentiation, which could exacerbate periodontitis.^147^ Furthermore, this study also found that Th17 cells derived from the intestinal microbiome could migrate to the oral cavity and induce oral inflammation.^147^ In addition, serum TNF-α and IL-17A, as well as IL-17A^+^ cells in the alveolar bone are significantly increased compared with the control group, which are reversed upon repairing of the gut barrier, indicating the influence of gut microbiota-related pro-inflammatory cytokine over-secretion.^85^

## Clinical applications

Considering that gut dysbiosis has a significant role in the pathogenesis of EIMs, several microbiota-based therapies have been proposed. For reduced commensal microbiota, medical supplementation is one of the treatment strategies. Direct replenishment of anti-inflammatory microbiota and indirect addition of beneficial metabolites or food ingredients are all acceptable options. On the other hand, for those increased harmful pathogens, antibiotics could be adopted. Fecal microbiota transplantation (FMT) may sometimes be considered if the gut microbiota is greatly disturbed. In the future, more individualized and accurate approaches are expected, including genetically modified micro-organisms, to eradicate the “criminal” pathogens or deliver anti-inflammatory factors in need. Clinical trials and animal experiments exploring microbiota-related treatments of EIMs are shown in Table 3.

**Table 3.** Microbiota-related animal experiments and clinical trials in the management of EIMs.

Disease	Subject	Intervention	Findings	Reference
**(A) Probiotics**				
Reactive arthritis	Salmonella Enteritidis-infected BALB/c mice	*Lactobacillus casei* (oral)	Consumption of *L. casei* reduced the bacterium invasiveness, degree of intestinal inflammation and synovitis, levels of TNF-α in knees and gut, levels of IL-17 in popliteal and mesenteric lymph nodes.	^148^
Rheumatoid arthritis	IFA and CII-immunized Wistar rats	*Lactobacillus casei* CCFM1074 (oral)	*L. casei* CCFM1074 regulated gut microbiota and unsaturated fatty acid metabolism, reducing arthritic symptoms, Th17 cells, plasma IL-6 and increasing Tregs in MLNs.	^149^
	CII-immunized HLA-DQ8 mice	*Prevotella histicola* (oral)	*P. histicola* produced AMPs and TJPs, significantly reduced intestinal permeability. *P. histicola* reduced Th17 responses, promoted production of Tregs and IL-10, significantly reducing incidence and severity of arthritis.	^150^
AS with quiescent UC	Patient (a pilot study, 18 patients)	*Lactobacillus acidophilus* and *lactobacillus salivarius* (oral, 4 weeks)	The probiotics reduced scores of Bath Ankylosing Spondylitis Disease Activity Index (BASDAI) and visual analogue scale (VAS).	^151^
Psoriasis	Imiquimod-induced BALB/c mice	*Lactobacillus pentosus* GMNL-77 (oral)	GMNL-77 significantly reduced pro-inflammatory cytokines in the skin, T cells for IL-17 and IL-22 production in the spleen, and areas of erythematous scaling lesions.	^152^
	Patient (a randomized, double-blind study, patients [26 psoriasis, 22 UC, 22 health])	*Bifidobacterium infantis* 35624 (oral, 6 weeks for UC and 8 weeks for others)	*B. infantis* 35624 significantly reduced TNF-α in psoriasis patients, IL-6 in UC patients and CRP in both.	^153^
	Patient (a randomized, double-blind study, 90 patients [45 probiotics + betamethasone +calcipotriol, 45 placebo + betamethasone + calcipotriol])	1:1:1 mixture of *Bifidobacterium longum* CECT 7347, *B. lactis* CECT 8145, and *Lactobacillus rhamnosus* CECT 8361 (oral, 12 weeks)	Probiotics significantly reduced Psoriasis Area and Severity Index at 6 weeks and the risk of relapse at 6 months.	^154^
Experimental autoimmune uveitis	IRBP, heat-inactivated MTB antigen and Pertussis toxin-immunized C57BL/6J mice	*Escherichia coli* Nissle 1917 (oral)	Probiotics promoted intestinal AMP production, prevented macrophage-induced inflammation and reduced T cell-mediated pro-inflammatory responses in extra-intestinal lymph nodes, eventually alleviating EAU.	^155^
	IRBP and Pertussis toxin-immunized C57BL/6 mice	IRT-5: *Lactobacillus casei*, *L. acidophilus*, *L. reuteri*, *Bifidobacterium bifidum*, and *Streptococcus thermophilus* (oral)	IRT-5 significantly reduced retinal histology score, percentage of CD8^+^ IL-17hi and CD8^+^ IFNγhi cells.	^156^
Periodontitis	Patients (a randomized, double-blind study, 41 patients [20 SRP + probiotics, 21 SRP + placebo])	*Bifidobacterium lactis* HN019 (oral, 30 days)	*B. lactis* HN019 promoted clinical, microbiological, and immunological benefits to the management of periodontitis.	^157^
	Patient (a randomized, double-blind study, 40 patients [20 SRP + probiotics, 20 SRP + placebo])	*Lactobacilli reuteri* (oral, 3 weeks)	*L. reuteri* significantly improved the plaque index, gingival index, bleeding on probing, and probing depth, and reduced the surgery risk.	^158^
**(B) Prebiotics**				
Arthritis	HLA-B27 transgenic rats	1:1 mixture of long-chain inulin-type fructans and short-chain inulin fraction oligofructose (oral)	Prebiotics significantly reduced the incidence rate of arthritis and colitis.	^159^
Periodontitis	Cotton ligature-treated Wistar rats	Mannan oligosaccharide (oral)	Prebiotics significantly protected against alveolar bone loss, reduced IL-10, IFN-γ, TNF-α and IL-1β, and further restored villous height and crypt depth.	^160^
**(C) Postbiotics**				
Arthritis	CFA and CII-immunized DAB/1J mice	Lithocholic acid (oral)	LCA significantly reduced arthritis score and pro-inflammatory cytokines.	^161^
	CFA-immunized Sprague Dawley rats	Indole-3-Carbinol (oral)	I3C significantly reduced clinical symptoms, ESR, TNF-α, IL-6 and histopathological changes. I3C protected the liver as well.	^162^
AS	Patients (36 patients)	Low starch diet (oral, 9 months)	A low starch diet reduced clinical symptoms, requirement of NSAIDs, seral levels of ESR and IgA.	^163^
Experimental autoimmune uveitis	IRBP, heat-inactivated MTB antigen and Pertussis toxin-immunized C57Bl/6J and Kaede transgenic mice IRBP and heat-inactivated MTB antigen-immunized B10.RIII mice	SCFAs (oral)	SCFAs significantly reduced uveitis severity in C57BL/6 mice. SCFAs increased Tregs in cervical lymph nodes, reduced Th1 and Th17 in mesenteric and cervical lymph nodes at 4 weeks. Propionate significantly reduced Th1 trafficking from gut to the spleen, and migration toward eyes tended to be reduced.	^120^
PSC	Patient (a randomized, double-blind phase II clinical trial, 161 patients, 102 with IBD)	NorUDCA (oral, 12 weeks)	NorUDCA significantly reduced ALP in a dose-dependent manner. Well tolerated.	^164^
	Patient (a randomized, double-blind phase II clinical trial, 76 patients, 43 with IBD)	Obeticholic acid (oral, 24 weeks)	OCA (5–10 mg) significantly reduced ALP in patients. The most common adverse event was pruritus.	^165^
	Patient (a randomized, double-blind phase II clinical trial, 52 patients, 31 with IBD)	cilofexor (oral, 12 weeks)	Cilofexor 100 mg significantly reduced ALP, GGT, ALT, AST, C4 and BAs. Well tolerated.	^166^
IBD-induced secondary liver injury	DSS-administered C57BL/6J mice	Milk fat globule membrane (oral)	MFGM reduced DSS-induced hepatic injury. MFGM improved gut barrier, and increased GST activity in the liver.	^167^
Periodontitis	Patient (a randomized, double-blind study, 36 patients [19 SPT + postbiotics, 16 SPT + placebo])	Heat-killed *Lactobacillus plantarum* L-137 (oral, 12 weeks)	The postbiotics decreased the depth of periodontal pockets more effectively.	^168^
	Ovariectomized Sprague-Dawley female rats	Berberine (oral)	Berberine significantly reduced alveolar bone loss.	^85^
**(D) Antibiotics**				
Ankle enthesitis (peripheral SpA)	DSS-administered SKG mice	Meropenem and vancomycin (oral)	Meropenem and vancomycin attenuated ankle enthesitis, decreased Th1 and Th17 cell levels in the spleen.	^169^
Experimental autoimmune uveitis	IRBP-induced B10.RIII mice	Metronidazole, vancomycin, neomycin, ampicillin (oral)	Both oral metronidazole and vancomycin alone reduced ocular inflammation significantly by increasing Tregs and decreasing Teffs. Gut microbial diversity clustering was associated with uveitis clinical scores.	^170^
PSC	Patient (a randomized, double-blind pilot study, 35 patients, 29 with IBD)	Vancomycin, metronidazole (oral, 12 weeks)	Both vancomycin and metronidazole decreased bilirubin, Mayo PSC risk score, etc. Only patients treated with vancomycin reached the primary endpoint [decrease in alkaline phosphatase (ALK) at 12 weeks], and with less adverse effects.	^171^
	Patient (a randomized, triple-blind clinical trial, 29 patients [18 vancomycin + UDCA, 11 placebo + UDCA], 21 with IBD)	Vancomycin (oral, 125 mg q6h, 12 weeks)	Vancomycin reduced clinical symptoms, ALP, GGT, ESR and Mayo PSC risk score significantly.	^172^
	Patient (a randomized, double-blind study, 80 patients [39 metronidazole + UDCA, 41 placebo + UDCA], 65 with IBD)	Metronidazole, UDCA (oral, 36 months)	Combination of MTZ and UDCA significantly reduced ALP and New Mayo Risk Score.	^173^
	Patient (a pilot study, 16 patients, 14 with IBD)	Minocycline (oral, 100 mg bid, 1 year)	Minocycline significantly reduced ALP and Mayo risk score, but not serum bilirubin or albumin. Well tolerated.	^174^
	Meta-analysis for patients with PSC		Vancomycin seemed as the most effective antibiotic with regard to clinical improvement and adverse effects.	^175^
	C57BL/6 mice, treated with fecal samples from a patient with PSC	Metronidazole, vancomycin (oral)	Vancomycin or metronidazole reduced the Th17 immune response.	^67^
Periodontitis	Meta-analysis for patients with periodontitis		Amoxicillin plus metronidazole were associated with the best clinical outcomes (including probing pocket depth, bleeding on probing, and clinical attachment level).	^176^
**(E) FMT**				
PSC	Patient (a pilot study, 10 patients, 10 with IBD)	A single FMT	FMT significantly reduced ALP (＜50%) in 3/10 patients at 24 weeks.	^177^

Abbreviations: UC, ulcerative colitis; EIMs, extra-intestinal manifestations; SpA, spondyloarthropathy; AS, ankylosing spondylitis; EAU, experimental autoimmune uveitis; PSC, primary sclerosing cholangitis; IgA, immunoglobulin A; ESR, erythrocyte sedimentation rate; CRP, C-reactive proteins; TNF-α, tumor necrosis factor-α; IFN, interferon; IL-6, interleukin 6; IL-10, interleukin 10; IL-17, interleukin 17; IL-22, interleukin 22; Tregs, regulatory T cells; Th1 cells, T helper type 1 cells; Th17 cells, T helper type 17 cells; Teffs, effector T cells; IFA, incomplete Freund’s adjuvant; CII, type II collagen; LCA, lithocholic acid; I3C, indole-3-carbinol; IRBP, interphotoreceptor retinoid-binding protein; MTB, Mycobacterium tuberculosis; UDCA, ursodeoxycholic acid; NorUDCA, norursodeoxycholic acid; OCA, obeticholic acid; DSS, dextran sodium sulfate; MFGM, milk fat globule membrane; MTZ, metronidazole; BAs, bile acids; AMPs, anti-microbial peptides; TJPs, tight junction proteins; ALP, alkaline phosphatase; GGT, gamma-glutamyltransferase; ALT, alanine transaminase; AST, aspartate transaminase; GST, glutathione-S-transferase; MLNs, mesenteric lymph nodes; NSAIDs, non-steroidal anti-inflammatory drugs; SRP, scaling and root planning; SPT, supportive periodontal therapy.

### Probiotics

According to the International Scientific Association for Probiotics and Prebiotics, probiotics are living micro-organisms that provide health benefits when consumed adequately.^178^ After being introduced into the human body, probiotics not only produce anti-inflammatory metabolites to down-regulate inflammatory factors such as IL-6, IL-12, TNF-α, and relevant signaling pathways such as the NF-κB pathway, but also aid in inhibiting the growth of pathogens, repairing the gut barrier, and regulating the differentiation and proliferation of naïve lymphocytes.^134^

In BALB/c mice, introduction of *Lactobacillus casei* prevents intestinal and articular inflammation, with down-regulation of IL-1β, IL-6, IL-17, IL-23, and TNF-α not only in the knees, but also in the mesenteric and popliteal lymph nodes.^148^ In addition, after supplementation with *Lactobacillus acidophilus* and *Lactobacillus salivarius* for 4 weeks, the Bath Ankylosing Spondylitis Disease Activity Index and visual analogue scale were improved in a pilot study involving 18 patients with active AS.^151^

*Bifidobacterium infantis* 35624 and a 1:1:1 mixture of probiotics (i.e., *B. longum* CECT 7347, *B. lactis* CECT 8145, *Lactobacillus rhamnosus* CECT 8361) either reduce pro-inflammatory TNF-α and plasma C-reactive protein or reduce the Psoriasis Area and Severity Index in patients after an 8- to 12-week course of treatment.^153,154^ In BALB/c mice treated with imiquimod, *Lactobacillus pentosus* GMNL-77 significantly reduced erythematous scaling lesions and mRNA levels of pro-inflammatory cytokines such as IL-23 and IL-27.^152^ Interestingly, supplement of *Bifidobacterium breve* CCFM683 effectively down-regulated keratin 16/17, IL-17, and TNF-α expression, improving psoriasis via regulating the FXR/NF-κB pathway and the keratinocyte proliferation.^179^

Moreover, a combination of *L. casei*, *L. acidophilus*, *L. reuteri*, *Bifidobacterium bifidum*, and *Streptococcus thermophilus* successfully reduced retinal histological scores in C57BL/6 mice immunized with interphotoreceptor retinoid-binding protein, an animal model of autoimmune uveitis.^156^

In the context of periodontitis, introduction of *Bifidobacterium lactis* HN019 or *Lactobacilli reuteri* significantly improved the clinical index of periodontitis, including reduced probing depth, less bleeding on probing, and lower surgical risk.^157,158^ Importantly, no serious adverse events were reported in the clinical trials or the animal experiments described above.

Furthermore, next-generation probiotics such as *F. prausnitzii* and *A. muciniphila* have been proposed, and their therapeutic efficiency for IBD has been confirmed. These probiotics reduce infiltrating macrophages, suppress NF-κB signaling pathways, reduce IL-8 production, and eventually down-regulate the severity of colitis.^51,82^ Considering the similar pattern of gut dysbiosis between IBD and common EIMs, the application of next-generation probiotics in the management of EIMs might also be promising.

### Prebiotics

As suggested by the International Scientific Association for Probiotics and Prebiotics, prebiotics refer to substrates that are selectively utilized by micro-organisms to bring health benefits.^180^ Microbial fermentation of prebiotics, such as inulin and oligofructose, generates metabolites (e.g., SCFAs) that further regulate the gut micro-ecological system and the immune response.^180^

In the context of SpA, oral administration of long-chain inulin and oligofructose has been reported to significantly reduce the incidence of colitis and arthritis in HLA-B27 transgenic rats.^159^

As for animal models of periodontitis, oral supplementation of mannan oligosaccharide successfully protects against alveolar bone loss, reduces the expression of IL-10 and IFN-γ, down-regulates levels of TNF-α and IL-1β, and notably restores intestinal villi as well as crypt depth.^160^

Notably，prebiotics owns widely acceptable safety profile with few serious adverse events reported.

### Postbiotics

Under the guidance of the International Scientific Association for Probiotics and Prebiotics, postbiotics refer to dead micro-organisms or their components that benefit the host, including SCFAs, SBAs, etc.^181^

In the context of SpA, direct exogenous supplementation with SCFAs attenuates arthritis severity in various animal models.^121^ Besides, oral administration of SCFAs also prevents the activation of effector T cells and the trafficking of immunocytes toward the spleen and cervical lymph nodes, ultimately down-regulating the severity of uveitis in C57BL/6J and Kaede transgenic mouse models.^120^

Research on PSC in this field is relatively abundant. Several phase II clinical trials have been launched with a course of 12–24 weeks, reporting that either norursodeoxycholic acid (a derivative of SBAs), obeticholic acid (an FXR ligand), or cilofexor (an FXR agonist) significantly reduce alkaline phosphatase in the serum of patients with PSC (with or without IBD).^164–166^ Furthermore, in C57BL/6J mice with IBD-related liver injury, the addition of milk fat globule membrane is associated with decreased pro-inflammatory cytokines, restoration of *Faccalibacumum* and *Roseburia*, attenuated colitis and liver injury, and re-activation of the glutathione transferase pathway.^167^

For patients with periodontitis, oral intake of heat-killed *Lactobacillus plantarum* L-137 has effectively reduced the depth of probing in patients who simultaneously underwent supportive periodontal therapy and had a depth no less than 4 mm at baseline.^168^ Moreover, berberine promotes butyrate production, improves the gut barrier, decreases circulatory LPS and pro-inflammatory cytokine levels, and down-regulates pro-inflammatory cells in alveolar bone, eventually ameliorating alveolar bone loss in animal models of periodontitis.^85^ Similarly, no severe adverse events have been reported in postbiotics.

### Antibiotics

Clinically, antibiotics are used to kill pathogenic bacteria or to inhibit their proliferation.

In the context of SpA, oral administration of meropenem and vancomycin effectively inhibits the development of peripheral enthesitis, accompanied by reduced Th1 and Th17 cells in the spleens of BALB/c and SKG mice.^169^ As for uveitis, metronidazole or vancomycin has been shown to down-regulate uveal inflammation and increase the abundance of Tregs in extra-intestinal lymphoid tissues of B10.RIII mice pre-treated with interphotoreceptor retinoid-binding protein.^170^ As for patients with PSC, application of vancomycin, metronidazole, and minocycline resulted in the improvement of liver enzymes and Mayo risk scores.^175^

Moreover, amoxicillin plus metronidazole, metronidazole alone and azithromycin have been reported to effectively improve clinical outcomes in patients with periodontitis, among which amoxicillin plus metronidazole performs the best to reduce probing pocket depth, bleeding on probing and improve clinical attachment level.^176^

### Fecal microbiota transplantation

Considering the significant involvement of the gut microbiota in the pathogenesis of EIMs, a complete reset of the microbiota would be an option. FMT refers to the therapy transplanting microbiota from healthy human feces into the alimentary tract of patients, enabling rapid restoration of the gut micro-ecologic system.^82^ However, FMT in the context of EIMs is rare and is mainly reported in patients with PSC (Table 3). Notably, the effect of FMT is disturbed by various factors, including the fecal quality of donors (e.g., proportion of SCFA-producing microbiota), the preparation procedure of fecal material, the administration approach and frequency, the individualized gut micro-organism composition before FMT (including bacteria, fungi, and viruses), and the operator techniques.^82^ Recently, enriched *Bacteroides* spp. (donor [D]), *Eubacterium hallii* (patient [P]), *Roseburia inulivorans* (P), reduced *Streptococcus* spp. (D), and up-regulated SCFAs and SBAs (P) have been correlated with better prognosis after FMT in patients with IBD.^182^ Given the similar pathogenesis between IBD and EIMs, possible correlations among such microbiota and the FMT effect in patients with various EIMs could be explored in further studies.

## Conclusions and perspectives

**Figure 2.**
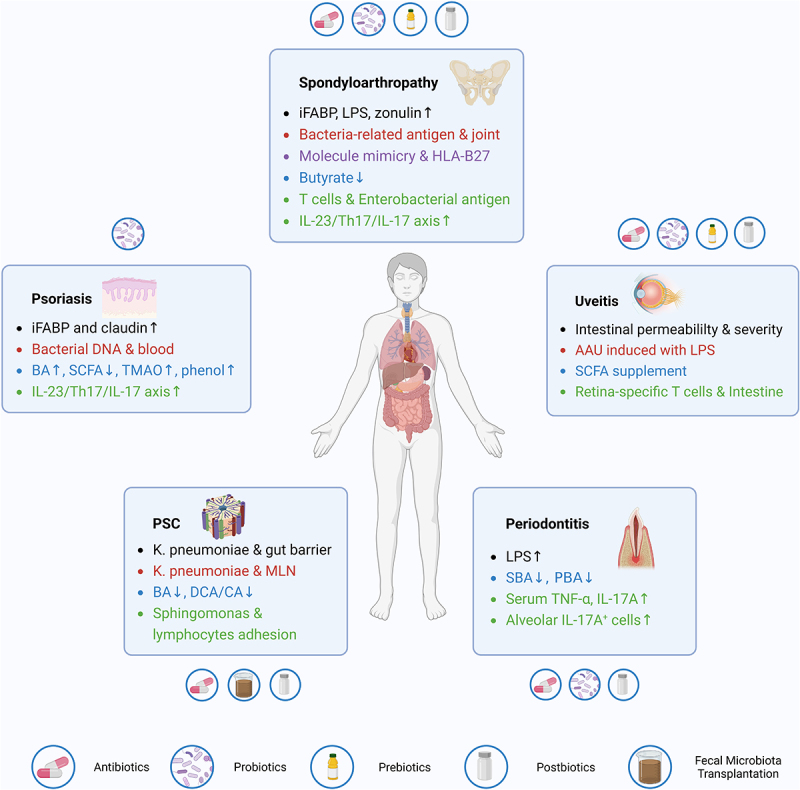
Summary of the roles of gut microbiota and relevant therapeutic applications in four common EIMs. EIM, extra-intestinal manifestation; PSC, primary sclerosing cholangitis; MLN, mesenteric lymph node; BA, bile acid; PBA, primary bile acid; SBA, secondary bile acid; DCA, deoxycholic acid; CA, cholic acid; AAU, acute anterior uveitis; LPS, lipopolysaccharide; SCFA, short-chain fatty acid; iFABP, intestinal fatty acid binding protein; TMAO, trimethylamine N-oxide; TNF-α, tumor necrosis factor-α; IL-17, interleukin-17.

In the context of IBD-associated EIMs, elevated mucin-degrading microbiota, pore-inducing microbiota, reduced SCFA-producing microbiota, and SBAs-transforming microbiota are all responsible for the impaired gut barrier, which could promote the translocation of gut microbiota, antigens, or toxins into extra-intestinal sites, including the blood, lymph nodes, synovial fluid, cutaneous tissues, liver, and oral cavity. Translocated microbiota can be engulfed and then presented by DCs and macrophages to promote lymphocyte activation and secretion of pro-inflammatory cytokines, especially IL-23 and IL-17. Moreover, certain bacterial antigens that share similar amino acid sequences or molecular structures with self-antigens, activate auto-reactive immunocytes targeting human tissues. In addition, dys-regulated microbiota-related metabolites, such as BAs, SCFAs, tryptophan, medium-chain fatty acids, and trimethylamine N-oxides modulate the activation of the NLRP3 inflammasome, secretion of inflammation-related cytokines, differentiation of naïve T cells toward Tregs or Th17, trafficking of lymphocytes into extra-intestinal sites, and in turn mediate the gut microbiota composition. Over-activation of the IL-23/Th17/IL-17 axis also takes an influential position in this process. Given the essential roles of microbiota, relevant therapies, including probiotics, prebiotics, postbiotics, antibiotics, and FMT, have been explored to optimize the prognosis for patients with EIMs. (Figure 2)

However, current research on EIMs is not always strictly limited to IBD. Abnormal changes in the gut microbiota and metabolites might not be found in patients with coincident IBD and EIMs. Furthermore, EIMs studies focusing on the functions of gut microbiota are confined mainly to SpA, psoriasis, PSC, and periodontitis, leaving the microbiota-related pathogenesis of other EIMs, such as erythema nodosum and pyoderma gangrenosum to be explored. Few animal experiments and clinical trials of EIMs have been conducted for microbiota-related therapies. Further studies are warranted for a clear and comprehensive understanding of the pathophysiology of IBD-associated EIMs to better guide clinical management. In addition, the development of precision medicine and gene-modifying technology would facilitate the identification of the gut microbiome of individuals and offer more effective, convenient, and safer therapeutic options.
